# An evaluation of the impact of a national Minimum Unit Price on alcohol policy on alcohol behaviours

**DOI:** 10.1093/pubmed/fdae288

**Published:** 2024-11-24

**Authors:** Gretta Mohan

**Affiliations:** Economic and Social Research Institute, Dublin D02 A021, Ireland; Department of Economics, Trinity College, Dublin D02 PN40, Ireland

**Keywords:** alcohol, alcohol consumption, health protection

## Abstract

**Background:**

In 2018, Scotland pioneered national legislation which set a Minimum Unit Price (MUP) of 50 pence (∼US$0.64, €0.59) per unit of UK alcohol sold (8 g/10 ml). To inform policy development, we examine the policy effect using the Alcohol Use Disorders Identification Test (AUDIT-C), employing longitudinal data for over 17 200 individuals.

**Methods:**

The effect of MUP on AUDIT-C scores is inferred by employing difference-in-difference regression. Pre- and post-intervention alcohol behaviours of individuals from Scotland are compared to a matched ‘control’ from England. Drinking at hazardous and harmful levels could be identified, as well as the frequency of alcohol consumption, number of drinks and heavy episodic drinking. Estimates adjust for demographic, socioeconomic and health characteristics. Potential inequalities by gender, age and household income are examined.

**Results:**

MUP led to an estimated 5.3% reduction in the number of drinks consumed on drinking occasions, though a statistically significant effect on overall reported AUDIT-C scores or drinking at hazardous levels was not detected, with few differential effects for subgroups.

**Conclusions:**

Differences in the findings of this research compared to other studies may be explained by differences in population coverage collected in the survey data, compared to more comprehensive, population-wide administrative data, as well as sample attrition.

## Introduction

Alcohol has long been established as a major contributor to the global burden of disease, injury and mortality, with significant economic and social costs. ^1–4^ Reducing the health and societal harms arising from alcohol use by lowering overall population alcohol consumption has been a key priority for policymakers.^5^^,^^6^ According to the theory of change,^7^ the establishment of a Minimum Price per Unit (MUP) of alcohol will reduce the amount of alcohol purchased and consumed. Minimum unit pricing is suggested to benefit those who drink at hazardous levels, including younger and heavier drinkers, as these groups tend to seek out the cheapest forms of alcohol to afford higher levels of consumption.^8–10^ Scotland, a nation with one of the highest levels of alcohol-related harm and mortality in Western Europe,^11^ pioneered the implementation of national legislation which sets a MUP on alcohol sales. In 2018, the Scottish government introduced a price floor on alcohol sold, setting a legally binding minimum price of 50 pence (∼US$0.64, €0.59) per UK unit of alcohol sold (unit (8 g/10 ml) of pure alcohol). The Australian Northern Territory followed suit later in 2018, Wales in 2020 and the Republic of Ireland in 2022, while other governments such as Northern Ireland, Switzerland and New Zealand consider such policies.^12^ Forms of minimum pricing on alcohol have also been in place in Canada and several eastern European countries for a number of decades.^13^

The impact of Scotland’s innovative national MUP policy merits scholarly examination to inform policy development in this area, which is of significant international interest. Existing evaluations of MUP have analysed household shopping data,^14–18^ alcohol-related hospital attendances,^19–22^ deaths attributable to alcohol consumption,^22^ food purchases^23^ and road accidents.^24^^,^^25^ The research presented in this paper uses longitudinal survey data to evaluate the impact of the Scottish MUP policy on individual’s reported alcohol behaviours, as captured by the Alcohol Use Disorders Identification Test (AUDIT-C), a three-item consumption subscale of the AUDIT 10-item metric of consumption and harm.^26^ AUDIT-C scores provide a reliable and validated alcohol screening tool,^27^ where threshold values identify those who drink at hazardous and harmful levels.^28–30^ The various components of AUDIT-C afford greater nuance in the analysis of the policy effect on self-reports of frequency of alcohol consumption, amounts of alcohol consumed on drinking occasions and frequency of heavy episodic drinking.

A study using household purchasing data found that MUP in Scotland led to an increase in the price of alcohol and a 7.6% reduction in the amount of alcohol purchased eight months after the policy introduction, where decreases were most significant for beer, own-brand spirits, high strength cider and among households in the lowest income quintiles.^14^ Alcohol price increases and purchasing decreases persisted over a longer observation period, and MUP in Wales also resulted in alcohol price rises and reduced purchases.^16^ For both Scotland and Wales, reductions in alcohol purchases were largely restricted to households that bought the most alcohol,^15^ and the policy shifted alcohol purchases from higher to lower strength products.^16^ The findings of reduced alcohol sales chime with evidence from two provinces in Canada,^17^^,^^18^ where Canadian evaluations examine changes in the *level* of minimum prices over time (as opposed to initial implementation).

An evaluation of MUP in Scotland three years after implementation reports a 3% net reduction in alcohol consumption per adult, driven by off-trade sales, though MUP did not result in changes to on-trade sales.^31^ A study which compared administrative population-level data from Scotland and England found that MUP in Scotland could explain a 13% reduction in deaths attributable to alcohol consumption and a 4% decrease in overall alcohol-attributable hospitalizations.^22^ Analysis of the data by disease classification revealed that the declines in deaths were driven by reductions in deaths from chronic causes, particularly alcoholic liver disease (−11.7%) and alcohol dependence syndrome (−23.0%). However, despite MUP, an increase in hospitalizations for acute outcomes wholly attributable to alcohol consumption was observed in the timeframe of analysis (+9.9%) and acute intoxication (+3.9%).

Other studies of alcohol-related health outcomes found no effect of MUP in Scotland on alcohol-related ambulance callouts,^32^ emergency department attendances,^19^ prescriptions for alcohol dependence,^33^ or alcohol dependence and health status of attendees of alcohol treatment centres in Scotland.^30^

Several important gaps remain in our understanding of the impact of this policy, which this paper aims to shed light on. The dataset employed for analysis, the UK longitudinal household survey, ‘Understanding Society’, provides an opportunity for a natural experiment evaluation with a more robust controlled longitudinal research design (where many extant studies are cross-sectional^34^). The data provides ‘individual-level’ information on alcohol behaviours, allowing a greater understanding of who was affected by MUP to a greater or lesser extent in a way that primary evaluation studies examining aggregate alcohol sales cannot provide such insights. The use of ‘Understanding Society’ as an alternative dataset also presents an opportunity to triangulate findings with existing evaluations. The alcohol consumption instrument recorded in this dataset, the AUDIT-C metric, represents an alternative outcome of study, providing insight as to how the impacts of the MUP policy may differ across different aspects of an individual’s drinking behaviours. For instance, the impact of MUP on occasions of heavy drinking, which may be less responsive to price changes^35^ has been relatively overlooked—this is important because the burden of alcohol is affected by whether alcohol is consumed in small doses over time or in larger doses on a few occasions.^36^

A final area that merits continued academic scrutiny is the effect of the policy on different groups of the population. For example, women, especially those drinking moderately, have been found to be somewhat more price sensitive to alcohol than men, and minimum pricing options that operate predominantly in the off-trade sector impact women more than men.^37^^,^^38^ Young drinkers typically have more limited budgets and are more price-conscious; thus, they tend to choose cheaper drinks,^39^^,^^40^ and so their levels of alcohol consumption may be greater affected by the implementation of the price floor. We note that while an evaluation found that MUP in Scotland did not negatively affect underage drinkers,^40^ relatively less is known about how MUP may affect different age groups through the life course. Therefore, this study examines the effect of MUP in Scotland across a range of age categories from youth (15–24 years) to older age (65 plus). As also demonstrated by other studies,^14^^,^^15^ there may be different effects of the policy across different household income categories. It has been found that while low-income households are not the predominant purchasers of alcohol overall, they are relatively more likely to purchase ‘off-trade cheap alcohol’,^41^ and thus it is predicted that among low-income drinkers, MUP would reduce their alcohol consumption the most.

## Methods

### Study selection

A quasi-experimental research design with observational data is employed. A difference-in-differences empirical strategy is adopted to assess the impact of the 2018 introduction of a MUP on alcohol sales in Scotland on alcohol consumption. Changes in alcohol consumption are compared in ‘treatment’ and ‘control’ groups between two-time points: ‘pre-policy’, where wave 7 of ‘Understanding Society’ collected data from households in a 24-month period across 2015–17, and ‘post-policy’, where wave 11 collected data for 2019–21 (Wave 7 is the first wave of **Understanding Society*,* which asks respondents the series of questions which make up the alcohol consumption instrument, AUDIT-C. This question is asked every two waves of ‘Understanding Society’*—*waves 8 (2016–18) and wave 10 (2018–20) do not have this AUDIT-C information. The sheer size of the survey means that it takes 24 months for a survey to cover all households once for each wave, and where the household is visited the following year for data collection, this interview falls under the subsequent wave. The bulk of interviews for wave 9 of ‘Understanding Society’ were conducted in 2018, the year in which the policy was introduced—in order to have uncontaminated before and after periods of analysis, waves 7 and 11 are selected as most appropriate for evaluation, particularly since the wording of the alcohol consumption question refers to the ‘previous 12 months’.). ‘Understanding Society’ began in 2009–10 as a longitudinal survey involving multiple components. The main survey involved a clustered and stratified probability sample of 24 000 households residing in Great Britain (England, Wales and Scotland) (for sampling details, see Lynn^42^), capturing baseline information on over 43 600 individuals. Interviews are carried out face-to-face in respondents’ homes by trained interviewers or through a self-completion online survey. Households recruited at the first round of data collection are visited for each wave on an annual basis to collect information on changes to their household and individual circumstances. Akin to many national longitudinal surveys, ‘Understanding Society’ has experienced sample attrition, whereby at wave 7, 51.9% of the original wave 1 sample was retained, and by wave 11, 39.9% remained.^43^ The individual response rates for waves 7 and 11 were 88 and 87%, respectively, where further details on samples can be found in the study technical reports.^43–45^

The treatment group comprises participants of ‘Understanding Society’ residing in Scotland, while the control group comprises participants residing in England. The analytical sample consists of those for whom we have data on for both periods of analysis, i.e. the individual’s examined have data for all variables studied for both the pre-and post-treatment periods. Individuals remain in the same group (treatment or comparator) throughout the sample period, and those moving in or out of the respective countries are excluded. Entropy balancing is carried out to match individuals in the treatment and control groups on their characteristics, and weights from this were applied to the estimation models.^23^ The unmatched and matched characteristics of the sample for the treatment and control group for the pre-treatment period are documented in Table 1. The analytical sample comprises 17 208 study participants with 34 416 observations (the derivation of the sample is detailed in Supplementary File Table S1, differences in the original sample for which there are observations with two waves of data compared to one wave in terms of household income categorization and alcohol consumption levels are documented in Supplementary Tables S2 and S3, while missingness in the analytical sample is documented in Supplementary Table S4).

**Table 1:** Descriptive statistics of the sample characteristics pre-MUP

*Variable (% or mean)*	*Treatment group (Scotland)*	*Control group-unweighted-(England)*	*Control group-weighted* *^a^* *-(England)*
Female	57.6	56.5	57.6
Age	51.5	49.2	51.5
Age category			
15–24 years	8.0	9.9	7.9
25–39	17.7	21.3	17.9
40–64	48.6	46.4	48.0
65+	25.8	22.3	26.3
Marital status			
Single	26.2	27.1	26.2
Married	54.6	57.2	55.6
Separated/divorced	13.0	10.7	13.0
Widowed	6.3	5.0	6.25
Highest qualification			
Degree or higher	28.2	30.1	28.1
Other higher level	15.4	12.6	15.4
A Level (upper secondary)	24.3	20.9	24.3
GCSE (lower secondary)	15.6	19.9	15.7
Other qualification	7.0	8.5	7.0
No qualification	9.6	8.0	9.6
Employment state			
Employed/self-employed	58.7	59.2	58.7
Unemployed	2.4	3.2	2.5
Retired	28.3	24.1	28.3
Student	4.1	5.5	4.1
Other	6.4	8.0	6.5
Gross monthly household income (£)	3876	4161	3947
Self-rated health			
Excellent/very good/good	83.6	82.6	83.6
Fair/poor	16.4	17.4	16.4
Smoker	13.6	13.1	13.6
Observations	1679	15 529	15 529

a The weights are those generated from entropy matching of the treatment and control groups.

The data can be accessed for research purposes via the UK Data Service. Since this paper involved analysis of anonymized secondary data, research ethics approval was not required, and it was not pre-registered ahead of undertaking the data analysis presented. The data collection for ‘Understanding Society’ is approved by the University of Essex Ethics Committee.

The primary outcome of study is a continuous variable from the AUDIT-C score. AUDIT-C is a continuous score of alcohol harm, ranging from 0 to 12, constructed from answers to the following three consumption-oriented questions:

**Table:** 

	*AUDIT-C questions*	*Responses (scoring system)*					
		*0*	*1*	*2*	*3*	*4*	*Score*
1	Thinking about the past 12 months, how often do you have a drink containing alcohol?	Never	Monthly or less	2–3 times per month	2–3 times per week	4+ times per week	
2	How many drinks do you have on a typical day when you are drinking?	1–2 drinks	3–4 drinks	5–6 drinks	7–9 drinks	10+ drinks	
3	How often have you had 6 or more units if female or 8 or more units if male, on a single occasion in the last year?	Never	Less than monthly	Monthly	Weekly	Daily or almost daily	
						Total score	

From this continuous score, a dummy variable of AUDIT-C score ≥5 = 1 is created, indicating drinking at a ‘hazardous level’ according to the UK government,^46^ zero otherwise. A dummy of AUDIT-C score ≥8 = 1 indicates drinking at a ‘harmful level’, zero otherwise. It is noted that while the definition of ‘hazardous’ and ‘harmful’ drinking adopted in this study is derived from the AUDIT-C threshold levels outlined, other definitions of hazardous and harmful drinking are of relevance for which we do not have data on. For example, Public Health Scotland^34^ defines hazardous drinking as that which increases an individual’s risk of harm, represented by a mean consumption of over 14 units of alcohol a week. Harmful drinking, on the other hand, is defined as a pattern of alcohol consumption causing mental and/or physical harm to health, represented by mean alcohol consumption of 35 units or more per week for women, and 50 units or more per week for men.

The scores on each of the items of the AUDIT-C score are also examined as outcomes. We note that for the data collected in the post-policy wave, the framing of the question with regard to the ‘previous 12 months’ requires careful treatment for responses collected in this period. MUP was introduced in May 2018, and, thus, responses collected on alcohol consumption in interviews carried out from May 2019 are valid, while those for the period January–April 2019 are dropped. It is also acknowledged that the outcome measure employed in this study is self-reported, relying on retrospective recall of alcohol behaviours, which can have issues in relation to incorrect recall or social desirability bias. Moreover, the AUDIT-C instrument is a shortened version of the original 10-item AUDIT assessment,^26^ which may be a relatively more blunt tool to assess changes in alcohol consumption, behaviour and alcohol-related problems.

The exposure of interest is a difference-in-difference variable which captures whether an individual is subject to MUP in Scotland for the post-policy period. Difference-in-difference ordinary least squares regression to estimate the effect of MUP may be represented as follows:


(1) \begin{align*} {Y}_{it}=&\ {\beta}_0+{\beta}_1{Scot}_i+{\beta}_2{MUP}_t+{\beta}_3\left({Scot}_i\ast{MUP}_t\right)\nonumber\\&+{\alpha}_i+{\lambda}_t+{\beta}_4{X}_{it}+{\varepsilon}_{it} \end{align*}

where


$$ {Scot}_i=1\ if\ Scotland\ (Intervention),0\ if\ England\ (Control) $$

\begin{align*} {MUP}_t=&\ 1\ time\ period\ where\ MUP\ implemented\ \left( post- policy\right),\\&0\ otherwise\ \left( pre- policy\right) \end{align*}

The primary dependent variable, ${Y}_{it}$, is the continuous AUDIT-C score of individual, $i$, at time, $t$ (secondary dependent variables include binary variables of hazardous and harmful drinking and continuous scores from the individual three items of the AUDIT-C score). The DID estimate, ${\beta}_3$, is given by the interaction of ${Scot}_i$ and ${MUP}_t$ and is the main variable of interest. It indicates the impact of being subject to MUP in Scotland during the policy period when both dummies equal one. Unadjusted crude DID effect estimates are first estimated (Model (1)), which control for individual fixed effects, ${\alpha}_i$, and time fixed effects, ${\lambda}_t$, as indicated by the interview month and year dummies to control for different drinking patterns across time and seasonality. Robust standard errors are employed, clustered on the individual identifier.

Then, in a second model (Model (2)), the DID estimates are further adjusted for potential confounders in examining alcohol behaviours arising from the demographic, socioeconomic, health characteristics and other health behaviours of respondents, ${X}_{it}$, represented by ${\beta}_4$. Specifically, the list of covariates encompasses gender, age, marital status, highest educational qualification, employment status, quintile of gross monthly household income, self-rated health and smoking.

To explore differential effects of MUP by subgroups, the difference-in-difference term is interacted with gender, age groups (15–24 years (our sample of underage drinkers (those aged 15–17 years) is not of sufficient size to allow for reliable statistical inference on this category and thus they are included in the ‘youth’ category), 25–39, 40–64 and 65 and above) and household income quintiles.

We note this analysis is limited by data constraints as there is no pre-policy AUDIT-C data recorded for participants of ‘Understanding Society’ prior to wave 7 to robustly examine parallel trends between the treatment and control groups. This is a limitation of our research, though we argue that England is an appropriate control since it has a relatively similar drinking and social culture, economy and population structure. In the period from 2012 to the implementation of MUP in Scotland in 2018, administrative data on alcohol-specific deaths report similar trends between Scotland and England.^34^ A majority of other controlled evaluations also employ England as the control group,^34^^,^^38^ and the two nations are unlikely to have been subject to differential exposures that would have differentially affected alcohol consumption trends across the timeframe of analysis.

To eliminate the possibility that cross-border shopping for alcohol^47^^,^^48^ could contaminate the results, a sensitivity analysis removes 60 study participants residing in Super Output Areas (SOAs) within 10 miles of the Scottish border with England, a neighbouring UK jurisdiction that is not subject to MUP. A Special Licence was granted by the UK Data Service to study the respondents at the finer-grained SOA geographical level (dataset SN 7248, project 223814).

## Results

### Descriptive statistics

**Fig. 1:**
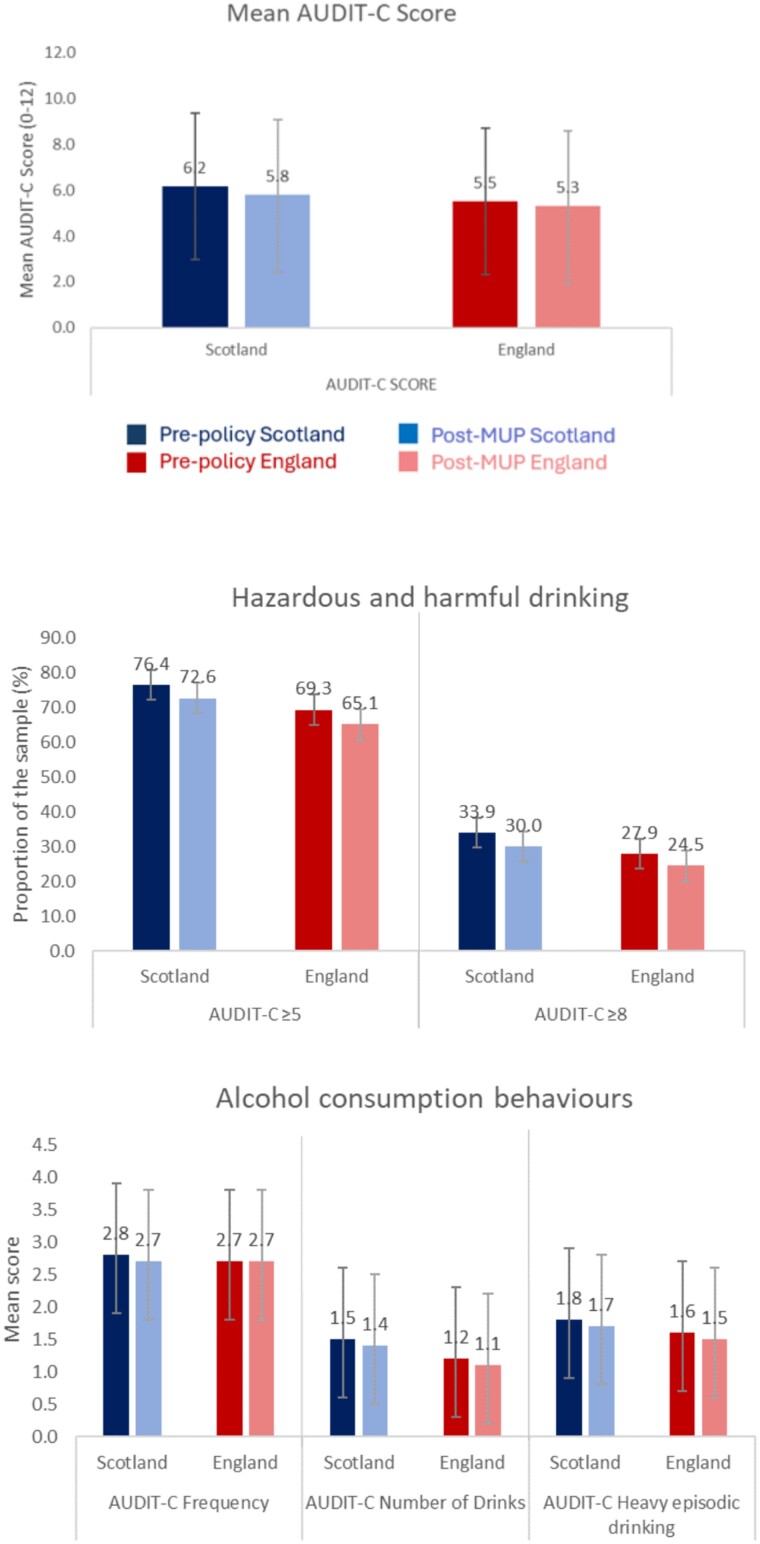
AUDIT-C scores, hazardous and harmful drinking variables, and AUDIT-C item scores of the treatment and control group, pre- and post- implementation of MUP in Scotland.

Figure 1 shows that the mean AUDIT-C score in the treatment group, Scotland, was higher than that of the control, England, and for both jurisdictions, the average score declined slightly between the pre- and post-MUP periods. Similarly, the proportion of the treatment group with AUDIT-C scores that indicate those drinking at hazardous and harmful levels was higher for Scotland, though decreased for both nations over the timeframe of analysis. For both groups, the frequency of alcohol consumption and heavy episodic drinking declined slightly. There was a slight decline in the average number of drinks consumed on drinking occasions in Scotland, though this was unchanged for England.

### Estimation results

The difference-in-difference estimation results reported in Table 2, do not demonstrate a statistically significantly discernible effect of MUP on the overall AUDIT-C alcohol consumption score, nor drinking at hazardous and harmful levels, though there was an estimated 5.3% (*P* = 0.023) reduction in the number of drinks consumed on a drinking occasion. Table 3 shows that where the difference-in-difference variable interacted with the variables representing the subgroups of interest, broadly, no statistically discernible effect of the policy on alcohol consumption behaviours is evidenced. However, the estimated reduction in the number of drinks consumed on a drinking occasion was significant for those in the middle quintile (an estimated reduction of the average number of drinks score by 0.21, *P* = 0.013), relative to the lowest income quintile; and the proportion of harmful drinkers in the middle income quintile was estimated to reduce by 8.4% (*P* = 0.035), relative to the lowest income quintile.

**Table 2:** Estimation results on AUDIT-C outcomes

*Outcome observations: 34 416; clusters: 17 208*	*AUDIT-C score*		*AUDIT-C ≥ 5*		*AUDIT-C ≥ 8*		*AUDIT-C frequency*		*AUDIT-C number*		*AUDIT-C heavy episodic*	
*Model*	*(1)*	*(2)*	*(1)*	*(2)*	*(1)*	*(2)*	*(1)*	*(2)*	*(1)*	*(2)*	*(1)*	*(2)*
Treatment (omitted from fixed effects estimation models as treatment group does not vary over time)												
Policy implementation	−1.553^***^ (0.520)	−0.068 (0.790)	−0.125 (0.088)	−0.008 (0.131)	−0.234^*^ (0.092)	0.024 (0.160)	−0.143 (0.239)	0.476 (0.361)	−0.329 (0.211)	−0.206 (0.326)	−1.080^***^ (0.218)	−0.338 (0.346)
Difference-in-difference (treatment^*^ Policy implementation)	**−0.028 [−0.143, 0.087] (0.633)**	**−0.024 [−0.139, 0.090] (0.058)**	**0.006 [−0.013, 0.024] (0.009)**	**0.006 [−0.013, 0.024] (0.009)**	**−0.010 [−0.032, 0.012] (0.011)**	**−0.009 [−0.031, 0.012] (0.011)**	**0.010 [−0.042, 0.062] (0.027)**	**0.012 [−0.040, 0.064] (0.026)**	−0.053^*^ [−0.099, −0.007] (0.023)	−0.053^*^ [−0.099, −0.007] (0.023)	**0.015 [−0.036, 0.066] (0.026)**	**0.017 [−0.034, 0.067] (0.026)**
Covariates												
Age		−0.277^**^ (0.103)		−0.023 (0.017)		−0.045^*^ (0.022)		−0.114^*^ (0.045)		−0.030 (0.041)		−0.133^**^ (0.048)
Marital status (reference category: single)												
Married/civil partner		−0.261 (0.169)		−0.050 (0.021)		−0.037 (0.029)		−0.014 (0.063)		−0.189 (0.073)		−0.058 (0.067)
Separated/divorced		−0.170 (0.165)		−0.037 (0.022)		−0.057^+^ (0.032)		−0.006 (0.063)		−0.162 (0.077)		−0.002 (0.073)
Widowed		−0.487^+^ (0.266)		0.078 (0.034)		−0.059 (0.042)		−0.086 (0.106)		−0.170 (0.098)		−0.230 (0.121)
Highest qualification (ref: degree or higher)												
Other higher level		0.410 (0.549)		0.011 (0.057)		0.066 (0.056)		0.102 (0.202)		0.169 (0.203)		0.143 (0.182)
A Level (upper secondary)		0.858^***^ (0.283)		0.060^*^ (0.030)		0.128 (0.045)		0.170 (0.116)		0.425^***^ (0.119)		0.270^**^ (0.100)
GCSE (lower secondary)		−0.320 (0.434)		−0.074 (0.049)		0.026 (0.068)		−0.264 (0.177)		0.044 (0.168)		−0.095 (0.148)
Other qualification		0.070 (0.556)		−0.056 (0.041)		0.090 (0.183)		0.022 (0.230)		0.073 (0.320)		−0.020 (0.179)
No qualification		−1.134^*^ (0.537)		−0.111^*^ (0.047)		0.053 (0.287)		−0.556^*^ (0.234)		−0.153 (0.250)		−0.418^**^ (0.159)
Employment state (ref: employed/self-employed)												
Unemployed		−0.280 (0.183)		−0.023 (0.023)		−0.014 (0.031)		−0.069 (0.076)		−0.096 (0.079)		−0.010 (0.034)
Retired		−0.119 (0.100)		−0.021 (0.019)		−0.021 (0.020)		−0.028 (0.051)		−0.010 (0.032)		0.014 (0.038)
Student/training/apprenticeship		−0.382^+^ (0.210)		−0.053^*^ (0.022)		−0.066^+^ (0.040)		−0.138 (0.093)		−0.124 (0.092)		0.030 (0.042)
Other/sick/home duties/maternity leave		−0.374^**^ (0.133)		−0.065^**^ (0.022)		−0.032 (0.025)		−0.206^***^ (0.056)		−0.100^+^ (0.059)		0.054 (0.044)
Quintile of household income (ref: quintile 1 lowest monthly income)												
Household income: q2		−0.043 (0.081)		−0.014 (0.014)		−0.014 (0.014)		−0.000 (0.037)		−0.032 (0.032)		−0.010 (0.034)
Household income: q3		0.007 (0.081)		0.014 (0.015)		−0.015 (0.016)		−0.017 (0.040)		0.010 (0.034)		0.014 (0.038)
Household income: q4		0.142 (0.096)		0.030 (0.016)		0.009 (0.018)		0.062 (0.045)		0.050 (0.038)		0.030 (0.042)
Household income: q5		0.133 (0.104)		0.003 (0.018)		0.007 (0.019)		0.028 (0.050)		0.050 (0.043)		0.054 (0.044)
Self-rated health (ref: excellent)												
Fair/Poor		0.086 (0.083)		0.015 (0.012)		0.002 (0.015)		0.038 (0.039)		0.015 (0.031)		0.034 (0.035)
Smoker		0.615^***^ (0.144)		0.041 (0.019)		0.080^**^ (0.023)		0.216^***^ (0.061)		0.157^**^ (0.060)		0.242^***^ (0.062)
Constant	6.435^***^ (0.277)	20.476^***^ (5.242)	0.749^***^ (0.051)	1.982^**^ (0.880)	0.382^***^ (0.055)	2.648^*^ (1.083)	2.767^***^ (0.128)	8.555^***^ (2.294)	1.541^***^ (0.117)	3.047 (2.089)	2.126^***^ (0.123)	8.868^***^ (2.453)
ρ rho: fraction of variance due to the individual	0.81	0.93	0.72	0.84	0.65	0.89	0.82	0.93	0.68	0.72	0.73	0.93

Level of statistical significance indicted as ^+^*P* < 0.10, ^*^P < 0.05, ^**^P < 0.01, ^***^P < 0.001. 95% confidence intervals in square brackets. Standard errors clustered on the individual identifier are presented in parentheses.

**Table 3:** Estimation results on AUDIT-C outcomes, subgroup interactions

*Outcome observations: 34 416; clusters: 17 208*	*AUDIT-C score*		*AUDIT-C ≥ 5*		*AUDIT-C ≥ 8*		*AUDIT-C freq*		*AUDIT-C number*		*AUDIT-C heavy episodic*	
*Model^*	*(1)^*	*(2)*	*(1)^*	*(2)*	*(1)^*	*(2)*	*(1)^*	*(2)*	*(1)^*	*(2)*	*(1)^*	*(2)*
DID interaction with female (Treatment Group^*^Policy implementation period^*^female) *(ref: male)*												
DID^*^female	0.046 (0.116)	0.071 (0.115)	0.023 (0.019)	0.025 (0.018)	0.005 (0.023)	0.008 (0.023)	−0.013 (0.053)	−0.004 (0.052)	0.003 (0.048)	0.010 (0.047)	0.055 (0.052)	0.066 (0.052)
DID interaction with age categories (Treatment^*^Policy implementation^*^age category) (ref: 25–64 years), adjusted for age												
DID^*^15–24 years	0.283 (0.379)	0.262 (0.350)	−0.041 (0.061)	−0.035 (0.059)	−0.078 (0.073)	−0.077 (0.071)	−0.110 (0.168)	−0.098 (0.165)	−0.388 (0.192)	−0.381 (0.184)	0.007 (0.168)	0.024 (0.160)
DID^*^25–40 years	−0.203 (0.227)	−0.187 (0.221)	−0.038 (0.055)	−0.034 (0.053)	−0.057 (0.063)	−0.057 (0.061)	−0.091 (0.148)	−0.084 (0.145)	−0.165 (0.174)	−0.161 (0.166)	−0.033 (0.149)	−0.022 (0.140)
DID^*^65 years	0.225^+^ (0.132)	0.223^+^ (0.132)	0.004 (0.057)	0.006 (0.055)	−0.009 (0.063)	−0.008 (0.062)	−0.045 (0.153)	−0.039 (0.150)	−0.080 (0.174)	−0.076 (0.166)	0.061 (0.153)	0.071 (0.223)
DID interaction with household income quintile (Treatment^*^Policy implementation^*^Household income quintile) ref: household income quintile 1)												
DID^*^Household income Q2	−0.192 (0.230)	−0.209 (0.229)	0.012 (0.036)	0.010 (0.038)	−0.007 (0.040)	−0.007 (0.040)	−0.028 (0.103)	−0.035 (0.103)	−0.172^+^ (0.089)	−0.174^+^ (0.089)	0.008 (0.101)	0.000 (0.100)
DID ^*^Q3	−0.330 (0.221)	−0.306 (0.220)	−0.033 (0.036)	−0.031 (0.036)	−0.087^*^ (0.040)	−0.084^*^ (0.040)	−0.064 (0.101)	−0.063 (0.101)	−0.226^*^ (0.085)	−0.212^*^ (0.085)	−0.040 (0.100)	−0.032 (0.100)
DID ^*^Q4	−0.259 (0.204)	−0.276 (0.202)	−0.051 (0.033)	−0.054 (0.033)	−0.049 (0.039)	−0.049 (0.039)	−0.036 (0.094)	−0.047 (0.093)	−0.162^+^ (0.087)	−0.164^+^ (0.086)	−0.060 (0.091)	−0.064 (0.091)
DID ^*^Q5	−0.371^+^ (0.192)	−0.366^+^ (0.191)	−0.048 (0.032)	−0.047 (0.032)	−0.065 (0.040)	−0.065 (0.040)	−0.090 (0.090)	−0.087 (0.090)	−0.141^+^ (0.077)	−0.143^+^ (0.077)	−0.140 (0.087)	−0.136 (0.087)

Note: In the case of the interaction term models displayed in Table 3, Model (1)^, the most basic specification models the group category, the DID terms and the interaction of the group category with the DID indicator. For brevity in reporting results, only the estimated result on the group term interacted with the DID indicator is displayed here. Model (2) includes all Model (1) variables, as well as the covariates outlined in Table 1. Full results are available on request from the corresponding author.

Level of statistical significance indicted as ^+^*P* < 0.10, ^*^P < 0.05, ^**^P < 0.01, ^***^P < 0.001.

Standard errors clustered on the individual identifier are presented in parentheses.

The sensitivity analysis results, reported in the Supplementary File (Table S5), revealed that the findings remained consistent when survey participants at the Scottish-English border were dropped from the analysis.

## Discussion

### Main finding of this study

The finding of a 5.3% reduction in the reported quantity of drinks consumed on drinking occasions revealed by the estimation of this research is consistent with literature, which infers that MUP in Scotland reduced alcohol consumption since it led to decreases in household purchases of alcohol.^14–16^ However, the study does not find strong statistically significant evidence of a reduction in overall AUDIT-C scores from the policy, nor for a reduction in the ‘proportion of’ those drinking at hazardous or harmful levels (A small average reduction in the ‘number of drinks on drinking occasions’ was observed for hazardous drinkers (−0.07 on the number of drinks scale), and harmful drinkers (−0.09) in Scotland over the analysis period (see Supplementary File Table S6). We note similarly that existing research findings paint a complex picture of the effect of the MUP policy in Scotland for heavier drinkers.^19^^,^^30^^,^^38^^,^^49^). The more muted findings of the research presented in this study, compared to the likes of studies that find a reduction in alcohol-related deaths and hospitalizations, including deaths of those with a diagnosis of alcohol use disorder,^22^ may be explained by differences in the population coverage achieved by the ‘Understanding Society’ surveys used in this analysis, which relies on respondent participation and engagement (where there may be non-engagement by those with the most severe alcohol problems^50^), compared to more comprehensive administrative data which captures records for the entire population. Furthermore, the data employed in this research is also subject to sample attrition from ‘Understanding Society,’ as previously mentioned in the *Methods* section and discussed below in the ‘Limitations of this study’ section.

MUP is intended to reduce health inequalities,^41^ where minimum prices are designed to have a more targeted effect on those who tend to purchase cheaper alcoholic beverages, particularly heavier drinkers on low incomes.^37^ This study found an indication of a relatively reduced number of alcoholic drinks for middle-income groups (third quintile) compared to the lowest-income group, which differs from studies that find that MUP legislation in Scotland precipitated greater improvements for the most socioeconomically deprived groups^34^ (e.g. reductions in deaths and hospitalizations wholly attributable to alcohol consumption^22^). For women, our finding of a lack of effect of MUP in this research also contrasts with other studies,^38^^,^^51^ though it may be explained by the tendency of females to purchase off-trade wine, which is less subject to price rises under MUP and has a lower own-price elasticity.^38^ MUP in Scotland was also not found to affect people of various age categories differentially in this study; this accords with an evaluation of MUP, which found little effect of the policy for underage drinkers,^40^ though we note the youth category of 15–24 years employed by this research is broader, encompassing young adults aged 18–24-year-olds who can legally drink alcohol both off- and on-trade. Among young adults, drinking in clubs and bars is a popular activity,^52^ characterized by higher alcohol prices, though pre-loading, i.e. the consumption of (cheaper) off-trade alcohol within private settings before socializing in licenced premises is also common,^53^ and thus the degree to which MUP may impact overall alcohol consumption on this age group is unclear.

### What is already known on this topic

The preponderance of studies using household purchasing data found that MUP lead to a reduction in alcohol purchases from off-trade retail outlets, particularly for the lowest income quintiles.^14–16^^,^^31^ An evaluation using administrative data on alcohol-attributable deaths found strong evidence of a reduction due to MUP, driven by decreases in deaths from chronic causes such as alcohol liver disease.^22^ Other studies, however, found no evidence that the policy brought about changes to alcohol dependence prescribing, alcohol-related ambulance callouts or ED attendances.^19^^,^^32^^,^^33^

There is conflicting evidence of the effect of MUP on those drinking at harmful levels and those with alcohol dependence.^30^^,^^49^^,^^54^ In summarizing research findings relating to these groups, Holmes^54^ highlights that household shopping studies found reductions in purchasing among higher alcohol purchasing households post-MUP.^14^^,^^15^ However, other studies using survey data of consumption diaries found no changes in the proportion of drinkers consuming at harmful levels.^30^ Other evidence suggests that harmful drinkers have been largely unresponsive to MUP price changes and increased their weekly spend on alcohol, cutting back on food and utilities or spending on other items, using food banks or charity, and borrowing from family and friends.^55^^,^^56^

The challenges of using natural experiment designs to evaluate public policy have been recognized in the case of the MUP in Scotland, and more broadly, in the public health discipline.^49^^,^^57^ Heterogenous studies using different datasets, samples and methodological approaches offer diverse and sometimes conflicting findings. Nevertheless, the variety of studies and their conclusions offer a rich source of insight into different consequences of the policy, identifying areas of success and, importantly, areas where additional support and better solutions are still required.

### What this study adds

Unlike the bulk of research published on this topic, this study examines whether the MUP policy in Scotland has had an impact on individual’s overall alcohol consumption behaviours using longitudinal, individual-level data, examining the AUDIT-C measure of alcohol consumption as the outcome of study from an alternative dataset. It affords an opportunity for research triangulation in evaluating the effect of MUP in Scotland, where findings across studies using multiple datasets and methodologies can be compared.

An advantage of the research presented in this paper is that it has been able to undertake sensitivity testing to explore whether there were differential effects of MUP in Scotland, where survey participants on the border with England were excluded and included from estimation. The results suggest there were little differential effects; substantiating claims that cross-border shopping on implementation of MUP was likely to be of a low and infrequent scale.^19^^,^^22^

In terms of policy implications from this work, the reduction in number of drinks consumed on drinking occasions attributable to MUP is extremely relevant to current and ongoing public policy debates about stricter alcohol control targeted at pricing. However, this study also did not find an impact of MUP on occasions of heavy drinking, which has not been extensively studied in the literature to date.

The results could suggest that a higher MUP may be required to better effect changes in drinking behaviours, which other countries considering the implementation of an MUP could implement from the start. Moreover, the cost-of-living crisis in 2022–23 has seen increases in the prices of most consumer goods such that the relative minimum price of 50 pence on alcohol sales is comparatively lower. MUP may need to adjust for inflation since the real value is reducing over time.^18^ However, these considerations must be balanced against any potential harmful consequences, such as greater financial strain experienced by those with alcohol dependence.^54^ Evidence suggests that attitudes to MUP on alcohol have become more favourable over time in Scotland,^58^ where it is felt that MUP could help tackle problems caused by alcohol; though for some, scepticism remains as to the effectiveness of the policy, particularly for those with alcohol dependence.

### Limitations of this study

‘Understanding Society’ did not collect AUDIT-C scores in the survey waves prior to the pre-policy baseline period, limiting the scope for a parallel trends analysis. Additionally, the self-reported answers to AUDIT-C could be subject to measurement error from under-reporting of alcohol consumption.^9^ The reasons for this may include poor recall or a desire to improve perceptions of oneself in an interview.^59^ The AUDIT-C is the best available metric in the dataset, though it may not be sensitive enough to pick up very small shifts in alcohol consumption induced by MUP to which further research could confirm or dispute. Furthermore, respondents of ‘Understanding Society,’ which comprise our longitudinal sample for study, may not sufficiently represent those who experience the most difficult problems with alcohol in society (where dependent drinkers, those on very low incomes, and the homeless are less inclined to participate and be retained, in social surveys^50^), and thus the estimated effect of MUP from this study may be biassed downwards. The issue of sample attrition in the dataset is also noteworthy; Supplementary Table S3 shows that there is a higher proportion of respondents with more hazardous drinking levels in the sample for which there are two waves of data compared to the sample for which only one wave of data is available. Since the analysis of this research relies on the data for two waves, it is possible that the composition of this sample may bias responses against finding less heavy levels of drinking post-policy.

The generalizability of these results may extend to other settings which adopt a low MUP. More research is required to inform the evidence base on the effectiveness and cost-effectiveness of MUP on alcohol intake and other potential outcomes, for example, financial strain, from a variety of jurisdictions. Where countries plan on introducing MUP, for instance, New Zealand, scholars and clinicians could collect baseline outcomes for which there is little evaluation evidence thus far, such as the more comprehensive AUDIT-10^60^ and CAGE scores.^61^

## Supplementary Material

Jan_Supplementary_File_MUP_fdae288

## Notes

**Gretta Mohan,** Senior Research Officer
