# Supplementary material for: An evaluation of the impact of a national Minimum Unit Price on alcohol policy on alcohol behaviours
Source: J Public Health (Oxf). 2024 Nov 24;47(1):e94–e105. doi: 10.1093/pubmed/fdae288 (PMC11879053; doi:10.1093/pubmed/fdae288)
Supplement: Jan_Supplementary_File_MUP_fdae288 [file jan_supplementary_file_mup_fdae288.docx]

**Supplementary File**

An evaluation of the impact of a national Minimum Unit Pricing on alcohol policy on alcohol behaviours

Note: all results are available from corresponding author upon request.

**Table S-1: Derivation of sample for analysis**

| Original *Understanding Society* sample | | | |
| --- | --- | --- | --- |
| Wave | Treatment | Control | Total |
|  | Scotland | England |  |
| Pre-MUP (7) | 3392 | 33087 | 36479 |
| Post-MUP (11) | 2604 | 24655 | 27259 |
| Total | 5996 | 57742 | **63738^±^** |
|  | | | |
| **Exclusions:** | | | |
| 1. Those who moved between Scotland & England between wave 7 & 11 | | | -157 |
| 2. Those who’s post-MUP interview (wave 11) occurred in months January-May 2019 because the framing of the AUDIT questions concern alcohol intake in the *previous 12 months*. MUP introduced in May 2018. | | | -4101 |
| 3. Those who do not occur in the sample for both waves of analysis | | | -20502 |
| 4. Those with missing observations on the outcome or exposure or covariate variables* (based on n=38978) | | | -2786 |
| 5. Those with full observations on variables of interest but only have 1 wave of data after exclusion step 4 | | | -1776 |
| **Analytical sample** | | | |
| Wave | Treatment | Control | Total |
|  | Scotland | England |  |
| Pre-MUP (7) | 1679 | 15529 | 17208 |
| Post-MUP (11) | 1679 | 15529 | 17208 |
| Total | 3358 | 31058 | **34416** |
| Sensitivity analysis exclusion: | | | |
| Those who live within 10 miles of the Scottish border | | | -60  (34356) |
| Notes: *Missingness table for variables of interest documented below | | | |

**^±^** We explored whether there is a differential in the original observation samples (the 63,738 observations sample before exclusions were applied) for which there was 2 waves of data for, compared to the observation sample for which there was only 1 wave of information for, in terms of income groups (quintiles of income) and heavier drinking levels (hazardous and harmful drinking). The tables below show that there was a slightly lower proportion of those in the lowest income quintile for the sample which has two waves of data (Table S-2). Table S-3 also shows that the sample with two waves of data also had higher representation from those engaged in hazardous drinking (AUDIT ≥5), and a very slightly higher proportion of those engaged in harmful drinking (AUDIT ≥8).

**Table S-2: Comparing samples for observations with 1 and 2 waves of data in terms of income quintiles (pre-exclusion)**

|  | Number of waves of data for observation (waves 7 & 11 *Understanding Society*) | |
| --- | --- | --- |
| **Quintile of household income** | **1** | **2** |
| **1** | 20.4% | 19.4% |
| **2** | 19.7% | 20.0% |
| **3** | 20.4% | 20.0% |
| **4** | 19.3% | 20.2% |
| **5** | 20.2% | 20.4% |
|  | 100% | 100% |
|  | Pearson chi2 (4) =12.5 | P=0.014 |
| Obs=63,738 | 17,658 | 46,080 |

**Table S-3: Comparing samples for observations with 1 and 2 waves of data in terms of alcohol consumption levels (pre-exclusion)**

|  | Number of waves of data for observation (waves 7 & 11 *Understanding Society*) | |
| --- | --- | --- |
| **AUDIT ≥5**  **(Hazardous drinking)** | **1** | **2** |
| **0** | 40.5% | 33.0% |
| **1** | 59.5% | 67.0% |
|  | 100% | 100% |
|  | Pearson chi2 (1) =278.1 | P=0.000 |
| Obs=58,608* | 14,877 | 43,731 |
|  |  |  |
| **AUDIT ≥8**  **(Harmful drinking)** | **1** | **2** |
| **0** | 74.0% | 73.1% |
| **1** | 26.0% | 26.9% |
|  | 100% | 100% |
|  | Pearson chi2 (1) =4.5 | P=0.033 |
| Obs=58,608* | 14,877 | 43,731 |
| *Lower sample size due to missing responses on AUDIT-C questions – see Table S-4 for information on missingness for the analytical sample | | |

**Table S-4: Details on missingness on observations of variables of interest**

| Variable | Missing | % Missing  (% of 38978* observations) |
| --- | --- | --- |
| AUDIT-C Score | 2016 | 5.2 |
| AUDIT-C Frequency | 1832 | 4.7 |
| AUDIT-C Number | 1936 | 5.0 |
| AUDIT-C Binge | 1895 | 4.9 |
| Sex | 0 | 0.0 |
| Age | 0 | 0.0 |
| Marital status | 943 | 2.4 |
| Highest educational qualification | 648 | 1.7 |
| Employment status | 48 | 0.1 |
| Gross household income | 10 | 0.0 |
| Self-rated health | 1777 | 4.6 |
| Smoker | 860 | 2.2 |
| *Total of 38978 from 63738 minus 157 minus 4101 minus 20502 as explained in sample size derivation Table S-1. | | |

**Table S-5: Sensitivity analysis results**

| Outcome | AUDIT-C Score | | AUDIT-C ≥ 5 | | AUDIT-C ≥ 8 | | AUDIT-C Freq | | AUDIT-C Number | | AUDIT-C Heavy episodic | |
| --- | --- | --- | --- | --- | --- | --- | --- | --- | --- | --- | --- | --- |
| Model | (1) | (2) | (1) | (2) | (1) | (2) | (1) | (2) | (1) | (2) | (1) | (2) |
| 1. SA 1: DID results from dropping residents 10 miles within the Scottish border with England | | | | | | | | | | | | |
| *Observations: 34356; Clusters: 17179* | | | | | | | | | | | | |
| Difference-in-difference  (Treatment*Policy implementation) | -0.019  (0.058) | -0.014  (0.058) | 0.005  (0.010) | 0.005  (0.009) | -0.009  (0.011) | -0.008  (0.011) | 0.014  (0.026) | 0.017  (0.026) | -0.052*  (0.024) | -0.052*  (0.024) | 0.019  (0.026) | 0.021  (0.026) |
|  |  |  |  |  |  |  |  |  |  |  |  |  |
|  |  |  |  |  |  |  |  |  |  |  |  |  |

**Table S-6: Difference in *average number of drinks* for those drinking at hazardous and harmful levels, pre- and post-MUP**

| Average number of drinks on a drinking occasion (see wording of question in p.9 of manuscript) | | | | | | | |
| --- | --- | --- | --- | --- | --- | --- | --- |
|  | Treatment (Scotland) | | | | Control (England) | | |
| (n=34,416) | | Pre-MUP | Post-MUP | *Diff* | Pre-MUP | Post-MUP | *Diff* |
| AUDIT ≥ 5 (n=23220) | | | | | | | |
| 1 | | 1.89 | 1.82 | *-0.07* | 1.61 | 1.57 | *-0.04* |
| 0 | | 0.42 | 0.39 | *-0.03* | 0.36 | 0.36 | *0* |
| AUDIT ≥ 8 (n=9282) | | | | | | | |
| 1 | | 2.52 | 2.41 | *-0.09* | 2.14 | 2.14 | *0* |
| 0 | | 1.05 | 1.00 | *-0.05* | 0.86 | 0.82 | *-0.04* |
